# Development of Room Temperature Stable Formulation of Formoterol Fumarate/Beclomethasone HFA pMDI

**Published:** 2009

**Authors:** D. Purohit, A. Trehan, V. Arora

**Affiliations:** Research & Development Centre (R&D I), Ranbaxy Laboratories Limited, Plot No. 20, Sector 18, Udyog Vihar Industrial Area, Gurgaon-122 015, India

**Keywords:** Impinger, aerosols, deposition of emitted dose, delivered dose uniformity

## Abstract

The primary aim of present investigation was to develop and formulate room temperature stable formulation of formoterol fumarate and beclomethasone dipropionate with extra fine part size of hydrofluoroalkane pressurized metered dose inhalers. Particle size distribution of hydrofluoroalkane pressurized metered dose inhalers was evaluated using Twin Stage Glass Impinger and Anderson Cascade Impactor. A tetrafluoroethane and/or heptafluoropropane were evaluated for preparation of hydrofluoroalkane pressurized metered dose inhalers. The fine particle fractions delivered from hydrofluoroalkane propellant suspension pressurized metered dose inhalers can be predicted on the basis of formulation parameters and is dependent of metering chamber of valve and orifice size of actuators. The results presented in investigation showed the importance of formulation excipients with formulation of pressurized metered dose inhalers viz, canister, valve and actuators used in formulations.

To develop room temperature stable formulation of formoterol fumarate/beclomethasone dipropionate hydrofluoroalkane (HFA) pressurized metered dose inhalers (pMDI) for lung delivery. The HFA pMDI were prepared from ozone friendly propellants viz, tetrafluoroethane and/or heptafluoropropane. A HFA based formulation of formoterol fumarate/beclomethasone dipropionate was developed to deliver 6 +100/200 μg per actuation over 120 doses. The present formulation comprises polyvinylpyrrolidone and/or polyethylene glycol in an amount sufficient to enhance the physical stability of suspension and provide extra fine particle size of formoterol fumarate/beclomethasone dipropionate. Formoterol fumarate and beclomethasone dipropionate have different modes of action. Beclomethasone dipropionate, a synthetic glucocorticoid given by inhalation at recommended doses has an antiinflammatory action, which plays an important role in the efficacy of beclomethasone dipropionate in controlling symptoms and improving lung function in asthma. Inhaled beclomethasone dipropionate probably acts topically at the site of deposition in the bronchial tree after inhalation. Formoterol is a selective β2 adrenergic agonist that produces relaxation of bronchial smooth muscle in patients with reversible airways obstruction. In addition to its bronchodilator action, formoterol also inhibits mast cell mediator release, plasma exudation and may reduce sensory nerve activation. Thus these two classes of drug address complementary aspects of the pathophysiology of asthma and COPD that neither drug class is able to achieve alone.

## MATERIALS AND METHODS

Excipients selected for evaluation were polyethylene glycol (at levels of 0.05 to 2.5% w/w, supplied by Clariant, India) and polyvinylpyrrolidone (at levels of 0.002 to 0.5% w/ w, supplied by Signet, India). The amount of micronised formoterol fumarate and beclomethasone dipropionate were mixed in a pressure vessel in a way to get the appropriate dose of formoterol fumarate and beclomethasone dipropionate to be able to deliver the desired doses to the patient. Beclometasone dipropionate/formoterol fumarate in above formulation is characterized by an extra-fine particle size distribution which results in a more potent effect than individual formulation of beclomethasone available in market. The above combination is indicated for regular treatment of asthma where use of a combination product (inhaled corticosteroid and long-acting beta_2_-agonist) is appropriate.

pMDIs were prepared by single step manufacturing process as given here: Introduction of active/excipients into pressure vessel, Addition of required quantity of propellant to provide sufficient number of doses, Filling of drug/propellant mixture into pre-crimped aluminum canister (supplied by Presspart, UK).

## RESULTS AND DISCUSSION

**Fig. 1:**
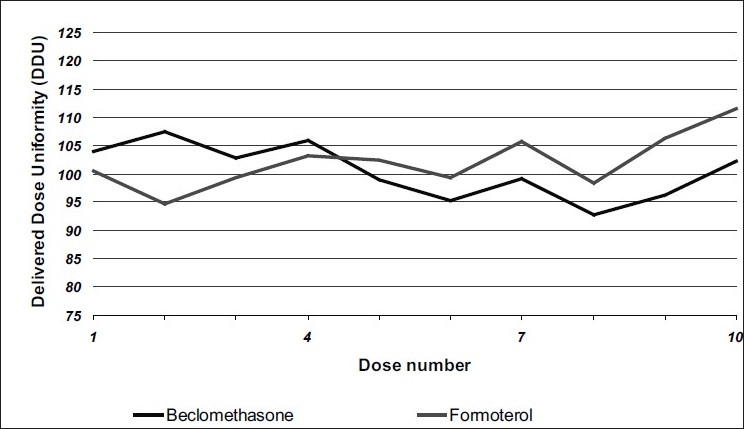
Delivered dose uniformity of the formulation using 0.3 mm actuator Delivered dose uniformity of the formulation containing (▬) formoterol and (▬) beclomethasone at initial conditions using a 0.3 mm actuator fitted on a DF316/50 valve.

**Fig. 2:**
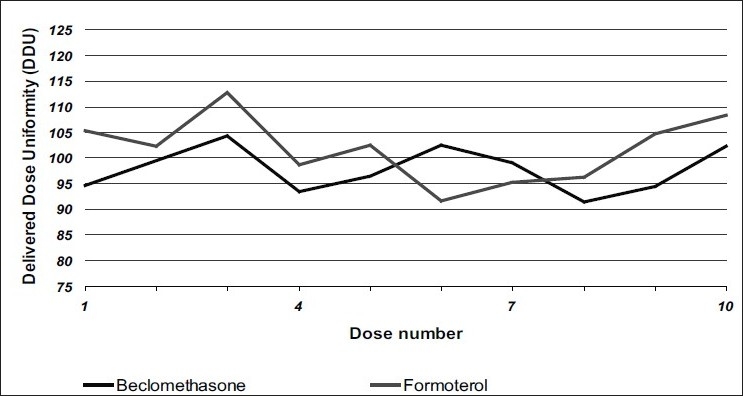
Delivered dose uniformity of the formulation using a 0.48 mm actuator Delivered dose uniformity of the formulation containing (▬) formoterol and (▬) beclomethasone at initial conditions using a 0.48 mm actuator fitted on a DF316/50 valve.

Delivered dose uniformity results obtained with a DF316/50 valve fitted with a 0.3 mm outlet orifice diameter actuator are shown in fig. 1 and those obtained with a DF316/50 valve fitted with a 0.48 mm outlet orifice diameter actuator are shown in fig. 2. Deposition of emitted dose by TSLI results are summarized in Table 1. During this study, Polyethylene glycol at levels less than 0.5% w/w was found to ensure good product performances and valve functioning throughout the MDI units' life. The level of polyvinylpyrrilodone selected also helped to decrease the settling of particles and prevents agglomeration of actives. This formulation was readily re-dispersible and avoiding invariability dosing of the drug. The stable suspension of particulate beclomethasone/ formoterol fumarate was selected by using HFA propellants closely matching the density of the micronised actives.

**TABLE 1:** DEPOSITION OF EMITTED DOSE BY TSLI

Drug	0.3 mm Actuator	0.48 mm Actuator
Beclomethasone dipropionate	40.23%	49.89%
Formoterol fumarate	56.81%	69.03%

Values are in terms of % of Label Claim

In conclusion, the novel combination of formoterol fumarate and beclomethasone dipropionate is a suspension based HFA pMDI product. The combined characteristics of room temperature stable formulation with good deposition of both drugs in lung provides unique characteristics to the above fixed dose combination, highly attractive in terms of patient usage requirements and commercial supply.
